# Coccidioidomycosis Complement Fixation Titer Trends in the Age of Antifungals

**DOI:** 10.1128/JCM.01318-18

**Published:** 2018-11-27

**Authors:** Ian H. McHardy, Bao-Tran N. Dinh, Sarah Waldman, Ethan Stewart, Derek Bays, Demosthenes Pappagianis, George R. Thompson

**Affiliations:** a Department of Medical Microbiology and Immunology, University of California, Davis, Davis, California, USA; b Department of Internal Medicine, Division of Infectious Diseases, University of California Davis Medical Center, Sacramento, California, USA; c Department of Infectious Diseases, Kaiser, San Diego, California, USA

**Keywords:** Coccidioides, coccidioidomycosis, complement fixation, dimorphic fungus, endemic mycoses, serology, valley fever

## Abstract

Coccidioidomycosis is associated with a broad spectrum of illness severity, ranging from asymptomatic or self-limited pulmonary infection to life-threatening manifestations of disseminated disease. Serologic studies before the widespread availability of antifungals established current understanding of serologic kinetics and dynamics. Chart histories and complement fixation (CF) titer trends were analyzed for 434 antifungal-treated coccidioidomycosis patients, who were classified by three infectious disease physicians as having either pulmonary uncomplicated coccidioidomycosis (PUC) (*n* = 248), pulmonary chronic coccidioidomycosis (PCC) (*n* = 64), disseminated coccidioidomycosis (DC) not including meningitis (*n* = 86), or coccidioidal meningitis (CM) (*n* = 36). The median maximal CF titers were 1:4 for PUC patients, 1:24 for PCC patients, 1:128 for DC patients, and 1:32 for CM patients. Approximately 25.4% of PUC patients, 6.2% of PCC patients, 2.3% of DC patients, and 8.3% of CM patients did not develop detectable titers during the study period. Maximal titers developed a mean of 31 days (95% confidence interval [CI], 13 to 50 days) after initial serologic positivity, with no significant differences between groups. Serologic recurrence occurred in 9% of PUC patients, 36% of PCC patients, 50% of DC patients, and 52% of CM patients. Median titer improvement rates were 91 days/dilution for PUC patients, 112 days/dilution for PCC patients, 136 days/dilution for DC patients, and 146 days/dilution for CM patients. Receiver operating characteristic (ROC) analysis revealed that CF testing retains moderate classification value for disseminated infections (area under the curve [AUC], 0.82 [95% CI, 0.78 to 0.87]) and complicated infections (AUC, 0.82 [95% CI, 0.77 to 0.86]). A suitable cutoff value for complicated infections is ≥1:32. Findings update serologic parameters that are relevant for clinical assessment of coccidioidomycosis patients in the triazole era.

## Abstract (precis)

Coccidioidomycosis is associated with a broad spectrum of illness severity, ranging from asymptomatic or self-limited pulmonary infection to life-threatening manifestations of disseminated disease. Serologic studies before the widespread availability of antifungals established current understanding of serologic kinetics and dynamics.

## INTRODUCTION

Coccidioidomycosis is a fungal disease caused by the dimorphic fungi Coccidioides immitis and Coccidioides posadasii. These soil-dwelling organisms exist in the western United States and Central and South America (1, 2). The diagnosis can be confirmed by microscopic demonstration of the characteristic forms (endosporulating spherules) in tissue or patient samples or by positive culture results (3). However, the majority of coccidioidal infections are identified and/or followed serologically by immunodiffusion and complement fixation (CF) testing (1). Enzyme immunoassays (EIAs) are available that can be used to screen for coccidioidomycosis in high-volume settings, with confirmation by immunodiffusion testing (1, 4). A typical course of infection involves IgM production to levels detectable by immunodiffusion within 1 to 3 weeks of symptom onset, followed shortly thereafter by IgG production (4, 5). The sensitivity of immunodiffusion, which can be used to detect both IgG and IgM antibodies, varies with the serum concentration, but the specificity likely exceeds 95% (6, 7). The CF test detects IgG antibodies to the chitinase antigen and is less sensitive than immunodiffusion (4, 7, 8).

The use of CF-based quantification of coccidioidal IgG in the care of coccidioidomycosis was described in the 1950s; results are predictive of disease prognosis (9), and the approach is useful in the evaluation of therapeutic responses to antifungal treatment (10). Despite widespread use of the CF test to monitor coccidioidal IgG antibodies, no prior study has systematically evaluated IgG antibody kinetics in patients receiving antifungal therapy. Studies performed in 1955 and 1956, in the preantifungal era, indicated that 82% of patients developed CF titers within 7 weeks of infection and 91 to 98% of patients with uncomplicated pulmonary infections developed maximal CF titers of ≤1:16 (5, 11). In comparison, only 12 to 23% of disseminated cases developed titers of ≤1:16. Following this initial observation, patients with titers of >1:16 were considered by some to warrant additional clinical and radiologic scrutiny for evidence of disseminated coccidioidomycosis (DC) (4, 7). However, serologic kinetics and dynamics have not been comprehensively described. The present study was designed to evaluate longitudinal CF titer dynamics in 434 patients who received antifungals for either pulmonary uncomplicated coccidioidomycosis (PUC), pulmonary chronic coccidioidomycosis (PCC), DC excluding meningitis, or coccidioidal meningitis (CM).

(These data were presented in part at the 2018 Coccidioidomycosis Study Group Meeting in Flagstaff, AZ.)

## MATERIALS AND METHODS

### Chart review.

Medical records were obtained for 605 coccidioidomycosis patients treated between 2009 and 2014 and were reviewed and adjudicated, according to type of coccidioidal infection, by at least two infectious disease physicians. A total of 434 patients met the inclusion criteria for this study. Inclusion criteria included ≥2 positive physician-ordered serologic immunodiffusion tests (more sensitive than CF and thus a better indicator of infection) performed in the reference laboratory. Exclusion criteria included pregnancy, breastfeeding, or <3 months postpartum at the time of diagnosis; immunosuppressive medications (including systemic corticosteroids, tumor necrosis factor alpha blockers, transplant medications, cyclophosphamide, azathioprine, methotrexate, or cyclosporine); phenotypic findings suggesting an underlying immunologic disorder (i.e., concurrent Mycobacterium avium complex or other opportunistic infections in addition to coccidioidomycosis); or HIV positivity. Cases were classified as either PUC (*n* = 248), with no evidence of coccidioidal relapse during 2 years of observation without antifungal therapy; PCC (*n* = 64), with continued symptoms and radiographic findings consistent with active pulmonary infection for >3 months, as described previously (12); DC (*n* = 86), with culture or histopathologic confirmation of extrathoracic disease; or CM (*n* = 36), with positive coccidioidal cerebrospinal fluid (CSF) serology in the context of CSF white blood cell (WBC), protein, and/or glucose changes suggesting infection (European Organization for Research and Treatment of Cancer/Invasive Fungal Infections Cooperative Group and the National Institute of Allergy and Infectious Diseases Mycoses Study Group definitions [3]) (Table 1). Patients who could not be categorized were excluded. All patients in this cohort received azole antifungal therapy. All study protocols were reviewed and approved by the University of California, Davis, institutional review board (protocol 233020-9).

**TABLE 1:** Study population

Characteristic	PUC	PCC	DC	CM
No. of patients	248	64	86	36
No. (%) of patients who developed maximal serum CF titers of ≥1:2	185 (74.6)	60 (93.8)	84 (97.7)	33 (91.7)
No. (%) of patients with titers resolved to <1:2 during study period	104 (56.2)	23 (38.3)	27 (32.1)	16 (48.5)
No. of serum specimens tested (mean [95% CI])	6.3 (5.7–6.7)	13.7 (11.7–15.6)^*a*^	19.8 (17.3–22.3)^*a*^	15.2 (10.5–20.0)^*a*^
Time between tested serum specimens (median [95% CI]) (days)	171 (144–198)	117 (92–141)^*a*^	116 (99–132)^*a*^	144 (97–191)
Duration of serologic follow-up monitoring (mean [95% CI]) (yr)	3.1 (2.8–3.5)	4.7 (3.8–5.6)^*a*^	8.9 (7.4–10.4)^*a*^	6.5 (4.9–8.1)^*a*^
Age (mean [range]) (yr)	46.9 (0.87–84.2)	49.2 (12.3–81.1)	42.1 (8.7–78.2)^*a*^	43.1 (19.9–68.7)
Gender (no. [%])				
Male	148 (60)	47 (73)^*b*^	69 (83)^*b*^	29 (81)^*b*^
Female	100 (40)	17 (17)^*b*^	14 (17)^*b*^	7 (19)^*b*^
Ethnicity (no. [%])				
Asian	5 (2)	1 (2)	3 (4)	1 (3)
African-American	5 (2)	6 (9)	41 (48)	9 (25)
Caucasian	29 (12)	6 (9)	8 (9)	8 (22)
Hispanic	83 (34)	26 (41)	14 (16)	6 (17)
Pacific Islander	0	0	7 (8)	0
Unknown/other	126 (51)	25 (39)	13 (15)	12 (33)

a Student's *t* test, *P* < 0.05, compared with PUC.

b Fisher's exact test *P* < 0.05, compared with PUC.

### Serologic data.

Serum coccidioidal immunodiffusion and CF titer data were collected for each enrolled patient for all specimens tested through June 2017. All patients had detectable coccidioidal CF (IgG) and/or coccidioidal precipitin (IgM) confirmed by immunodiffusion at least twice during the infection course, as well as at least 2 coccidioidal CF tests (not necessarily positive). Immunodiffusion and CF were performed as described previously (4). Titer analyses involved serum CF data or, when CF yielded invalid or equivocal results, quantitative immunodiffusion data. References to serologic titer data in this article refer to CF titer data unless indicated otherwise. Spent coccidioidal culture supernatant, prepared as described previously, was used as the antigen for immunodiffusion and CF assays (7).

### Data analysis.

All statistical, mathematical, and graphical analyses were performed in R, using custom scripts (13). Statistical analyses and comparisons involving titers were performed on log_2_-transformed titers; where necessary, results were converted back to titers by calculating 2^*x*^, where *x* is the log_2_-transformed titer. Histogram density comparison plots were generated using the R sm package (14). Receiver operating characteristic (ROC) plots and associated sensitivity, specificity, positive predictive value (PPV), area under the curve (AUC), confidence interval (CI), and Delong's significance analyses were performed using the R pROC package (15). The prevalence of complicated or disseminated disease for PPV calculations was the prevalence in the study population, which may not reflect proportions that are epidemiologically observed. Youden's *J* statistic was calculated to identify optimal classification titer thresholds (16).

### Titer trend plots.

**FIG 1:**
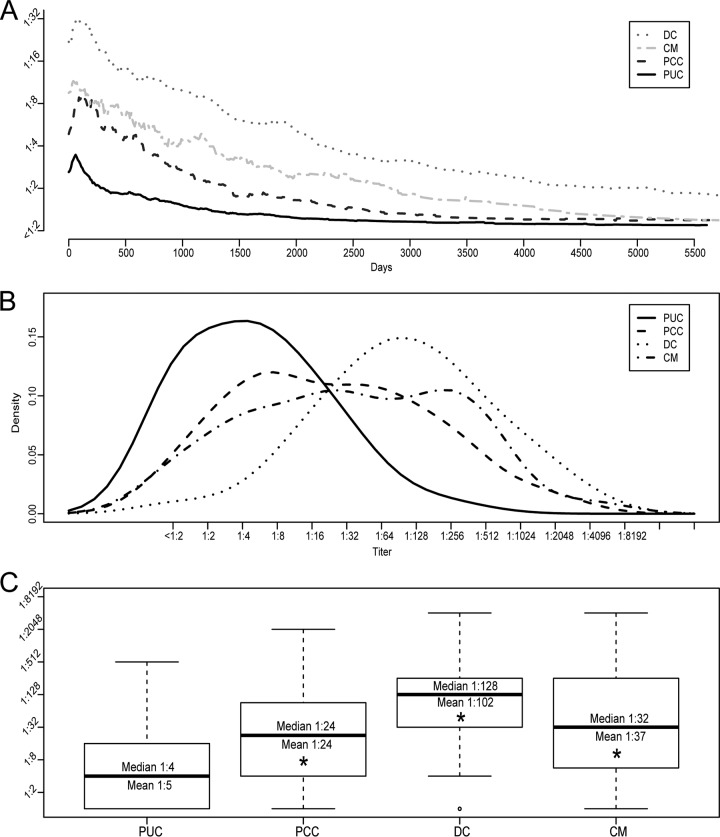
Titer trends and maximal titers. (A) Average titer trend for each group. (B and C) Histogram density plot (B) and boxplot (C) of maximal titers for each group. The average maximal titers shown in panel A differ from the results calculated for panel C due to temporal synchronization required for visualization and interindividual variations in the time to maximal titer. Boxes represent the first and third quartiles. Whiskers represent 1.5 times the interquartile range. *, Student's *t* test, *P* < 4.7 × 10^−6^.

The irregularity of sample numbers and intervals among patients and groups required *in silico* CF titer resampling with 10-day intervals for all patients. Resampling was performed by assigning CF titers at 10-day intervals (which we found facilitated an optimized granular view of overall trends) after the initial positive serologic test by replicating the closest previous titer. Titers were resampled up to a maximum of 5,500 days (∼15 years). For patients who lacked serologic data of this duration, titers were linearly imputed using the slope calculated as described below (serologic improvement rate). Each patient's titer history was synchronized using the date of the initial positive serologic test (the initial positive test was often an immunodiffusion test, given the relatively increased sensitivity of immunodiffusion testing, compared with CF) as day 0. Figure 1A presents intragroup average titers at 10-day intervals using these data.

### Serologic improvement rates.

Titer reduction rates among different types of infections were determined by calculating titer slopes for each patient using the following formula: *m* = −(Δtime/ΔCF), where ΔCF refers to the difference in log_2_-transformed maximal and lowest subsequent minimal CF titers and Δtime refers to the number of days between the maximal titer and the subsequent minimal titer.

### Serologic recurrence detection.

Serologic recurrence, defined here as a ≥4-fold increase (≥2 dilutions) in CF titer at least 90 days after initial serologic positivity, was bioinformatically detected and quantified using a custom R-based algorithm that identified local maxima in each patient's longitudinal titer history and removed any maxima that did not involve a ≥4-fold increase from either the previous titer or an immediately preceding local minimum.

## RESULTS

### Study population.

The study included 434 patients, including 248 with PUC, 64 with PCC, 86 with DC, and 36 with CM (Table 1). The average age at initial diagnosis was 46.9 years (range, 0.87 to 84.2 years) for PUC, 49.2 years (range, 12.3 to 81.1 years) for PCC, 42.1 years (range, 8.7 to 78.2 years) for DC, and 43.1 years (range, 19.9 to 68.7 years) for CM. All patients in this study received antifungal therapy; all PUC patients were treated with fluconazole. CF titer data yielded an average of 3.1 years (95% CI, 2.8 to 3.5 years) of serologic data for PUC patients, 4.7 years (95% CI, 3.8 to 5.6 years) of serologic data for PCC patients, 8.9 years (95% CI, 7.4 to 10.4 years) of serologic data for DC patients, and 6.5 years (95% CI, 4.9 to 8.1 years) of serologic data for CM patients. The average number of specimens serologically tested (by both immunodiffusion and CF) per patient during these periods was 6.3 specimens (95% CI, 5.7 to 6.7 specimens) for PUC patients, 13.7 specimens (95% CI, 11.7 to 15.6 specimens) for PCC patients, 19.8 specimens (95% CI, 17.3 to 22.3 specimens) for DC patients, and 15.2 specimens (95% CI, 10.5 to 20 specimens) for CM patients. The median number of days between serologic tests was 171 days (95% CI, 144 to 198 days) for PUC patients, 117 days (95% CI, 92 to 141 days) for PCC patients, 116 days (95% CI, 99 to 132 days) for DC patients, and 144 days (95% CI, 97 to 191 days) for CM patients. There were more male patients than female patients in all groups.

### Maximal serum CF titers and trends.

The average temporal titer trends for patients in each group (Fig. 1A) showed that the average patient developed a maximal CF titer shortly after initial serologic positivity and improved thereafter. Importantly, the maximal titers shown in Fig. 1A do not reflect the true average maximal titers in each group, because titers were temporally synchronized to simplify visualization. The maximal serum CF titer was then determined for each patient (throughout the infection course), and results were compared in aggregate between groups. Importantly, not all patients developed a titer detectable by CF. Approximately 25% of PUC patients (63/248 patients), 6% of PCC patients (4/64 patients), 2% of DC patients (2/86 patients), and 8% of CM patients (3/36 patients) failed to develop a detectable CF titer during the study period and were instead diagnosed and monitored by qualitative immunodiffusion, which is more sensitive and specific than CF for coccidioidal antibody detection. Thus, immunodiffusion remains important for the diagnosis and, in some cases, monitoring of coccidioidomycosis, particularly for patients who fail to develop a detectable CF titer or whose titer improves to <1:2 (4).

The distribution of maximal titers in each group is shown in Fig. 1B. Notably, PCC cases and CM cases had broader peak distributions, suggesting a range of disease severity, while DC and PUC cases had much tighter peak distributions. The median maximal titers were 1:4 (mean, 1:5 [95% CI, 1:4 to 1:6]; range, <1:2 to 1:512) for PUC cases, 1:24 (mean, 1:24 [95% CI, 1:15 to 1:39]) for PCC cases, 1:128 (mean, 1:102 [95% CI, 1:70 to 1:148]) for DC cases, and 1:32 (mean, 1:37 [95% CI, 1:18 to 1:75]) for CM cases (Fig. 1C). The increased mean titers observed for PCC, DC, and CM patients, compared with PUC patients, were statistically significant (Student's *t* test, *P* < 5 × 10^−6^), further solidifying the highly significant correlation between CF titers and disease severity.

To contextualize maximal titers observed in this study, CF titer data were extrapolated from published preantifungal studies for comparison (5). Patients with uncomplicated disease from the preantifungal era developed a median maximal titer of 1:2 (mean, 1:4 [95% CI, 1:4.2 to 1:4.5]), significantly lower than that of the PUC group (Student's *t* test, *P* = 0.032) but still within the 1-dilution (2-fold) error range of the method. Further analysis revealed that 83% (207/248 cases) and 94% (233/248 cases) of PUC cases developed maximal titers of ≤1:16 and ≤1:32, respectively (Table 2). In contrast, 91 to 98% and 97 to 99% of uncomplicated cases in the preantifungal studies developed titers of ≤1:16 and ≤1:32, respectively (5). These findings suggest that maximal titers of uncomplicated coccidioidomycosis cases are slightly higher in the antifungal era, compared with the preantifungal era. Meanwhile, 50% (32/248 cases), 20% (17/64 cases), and 42% (15/86 cases) of PCC, DC, and CM cases, respectively, developed maximal titers of ≤1:16, largely overlapping with disseminated case titers from preantifungal studies. This finding suggests that significantly higher titers are observed among some nondisseminated cases in the antifungal era, which could complicate classification accuracy of CF for disseminated cases.

**TABLE 2:** Maximum serum titer associations

Study and titer	No. (%) of cases			
	PUC	PCC	DC	CM
Current study				
≤16	207 (83)	32 (50)	17 (20)	15 (42)
≤32	233 (94)	39 (61)	25 (29)	20 (56)
Study by Smith et al. (5)				
≤16	(91–98)	NT^*a*^	(12–23)	(41–62)
≤32	(97–99)	NT	(27–40)	(75–81)

a NT, not tested explicitly.

### Quantitative analysis of CF titers as classifiers of coccidioidomycosis severity.

**FIG 2:**
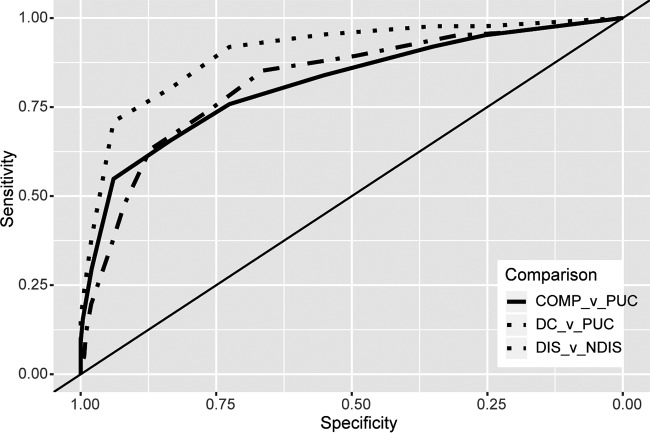
ROC plots. Overlaid ROC plots show the classification accuracy for DC versus PUC cases (AUC, 0.903 [95% CI, 0.86 to 0.94]) (a perfect classifier would have a sensitivity and specificity of 1 along all points and thus would have an AUC of 1), complicated (COMP) versus uncomplicated cases (AUC, 0.815 [95% CI, 0.77 to 0.86]), and disseminated (DIS) versus nondisseminated (NDIS) cases (AUC, 0.822 [95% CI, 0.78 to 0.87]). The *x* axis is reversed and is labeled “specificity,” as opposed to “1 − specificity.”

To begin examination of the extent to which CF titers can differentiate uncomplicated cases from nonmeningeal disseminated infections, ROC curves comparing DC and PUC patients were generated (Fig. 2, DC versus PUC comparison). The AUC was 0.903, indicating that CF can, if other disease manifestations are excluded, fairly reliably distinguish DC patients from PUC patients. Given the broad overlap of maximal titers observed among DC, PCC, and CM patients (Fig. 1), this pairwise comparison is clearly overly simplified but may align with preantifungal-era classification accuracy. Therefore, to define modern classification accuracy for disseminated (CM and DC) and nondisseminated (PUC and PCC) cases, cases were grouped accordingly and analyzed by ROC plot (Fig. 2, disseminated versus nondisseminated comparison). The AUC of the ROC curve was 0.822 (95% CI, 0.78 to 0.87), significantly (Delong's test, *P* = 0.0078) lower than the AUC of 0.903 for the pairwise disseminated disease comparison but still indicating moderate classification accuracy (17). To determine CF classification accuracy for complicated versus uncomplicated cases, all non-PUC patients (PCC, DC, and CM patients) were combined into a single group (complicated) and compared against PUC patients (Fig. 2, complicated versus PUC comparison). The AUC of this ROC curve was 0.815 (95% CI, 0.77 to 0.86), not significantly different from and nearly superimposing that for the disseminated versus nondisseminated comparison. To identify classification accuracy at each possible CF threshold, the sensitivity, specificity, and PPV were calculated at each titer between 1:2 and 1:1,024 for each relevant comparison. These data, shown in Table 3, revealed that no cutoff value with perfect sensitivity and specificity exists. Higher titers are more specific but less sensitive for disseminated and complicated infections, due to the small numbers of DC, CM, and PCC cases with relatively low CF titers.

**TABLE 3:** Sensitivity and specificity at each possible serum CF titer threshold

Titer threshold	Disseminated vs nondisseminated			Complicated vs uncomplicated		
	Sensitivity (% [95% CI])	Specificity (% [95% CI])	PPV (% [95% CI])	Sensitivity (% [95% CI])	Specificity (% [95% CI])	PPV (% [95% CI])
≥1:2	95.9 (91.8–99.2)	21.5 (17.3–26)	32.3 (30.9–33.7)	95.2 (91.9–97.8)	25.4 (20.2–31)	48.9 (47–51)
≥1:4	95.1 (91–98.4)	30.8 (26–35.9)	35 (33.1–37)	91.9 (88.2–95.7)	35.1 (29–41.5)	51.5 (49–54)
≥1:8	89.3 (83.6–94.3)	49.4 (43.9–55.1)	40.8 (37.9–44)	83.9 (78.5–89.2)	55.2 (48.8–61.3)	58.4 (54.7–62.2)
≥1:16^*a*^	85.2 (78.7–91)	66.3 (61.2–71.5)	49.8 (45.7–54.4)	75.8 (69.4–81.7)	72.6 (66.9–78.2)	67.4 (62.4–72.3)
≥1:32^*b*^	73.8 (65.6–82)	76.6 (71.8–81.4)	55.4 (49.4–60.9)	65.6 (58.6–72)	83.5 (79–87.5)	74.7 (69.2–80.7)
≥1:64	63.1 (54.9–71.3)	87.2 (83.3–91)	66 (58.8–73)	54.8 (47.8–61.8)	94 (90.7–96.8)	87.3 (81.6–92.8)
≥1:128	47.5 (39.3–56.6)	92 (89.1–94.9)	70 (61.5–79)	39.8 (32.8–46.8)	96.4 (94–98.4)	89.3 (82–95.2)
≥1:256	35.2 (27–43.4)	94.6 (92–97.1)	71.8 (61–82)	29.6 (23.1–36)	98 (96–99.6)	92.1 (84.2–98.2)
≥1:512	19.7 (13.1–27)	98.1 (96.5–99.4)	80.6 (65.4–93.1)	15.6 (10.8–21)	99.6 (98.8–100)	96.9 (88.4–100)
≥1:1,024	11.5 (5.7–17.2)	99 (97.8–100)	83.3 (61.5–100)	9.1 (5.4–13.4)	100 (100–100)	100 (100–100)

a Optimal Youden threshold for distinguishing disseminated from nondisseminated infections (*J* = 0.52).

b Optimal Youden threshold for distinguishing complicated from uncomplicated infections (*J* = 0.49).

To identify CF titer cutoff values with maximal classification performance for disseminated and complicated infections, Youden's *J* statistics (16) were calculated at each titer threshold. Using this method, the best performing CF classification threshold for disseminated versus nondisseminated disease was ≥1:16 (*J* = 0.52), with a sensitivity of 85.2% (95% CI, 78.7 to 91%), a specificity of 66.3% (95% CI, 61.2 to 71.5%), and a PPV of 49.8% (45.7 to 54.4%), indicating that ∼50% of patients with titers of ≥1:16 did not have disseminated infections. The best performing classification threshold for complicated versus uncomplicated cases was ≥1:32 (*J* = 0.49), with a sensitivity of 65.6% (95% CI, 58.6 to 72%), a specificity of 83.5 (95% CI, 79 to 87.5%), and a PPV of 74.7 (95% CI, 69.2 to 80.7%). Comparing PPVs between the cutoff values showed that the ≥1:32 threshold for complicated infections was more specific and reliable than the ≥1:16 threshold for disseminated disease.

### Time to maximal CF titers and serologic recurrences.

**FIG 3:**
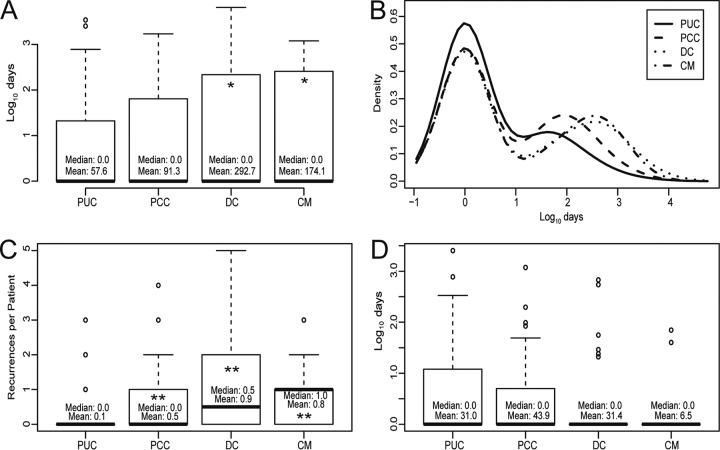
Maximal titer development kinetics. (A and D) Boxplots showing time to maximal titer for each group, including serologic recurrences (A) and excluding serologic recurrences (D). (B) Histogram density plot of time to maximal titer for each group, showing numerous outliers in each group, ranging from 10^2^ days to >10^3^ days. Outliers were more abundant for more complicated infections. (C) Number of recurrences per patient in each group, which contributed to the overestimated time to maximal titers in panel A. Titer data for boxplot analyses were log_10_ transformed to account for numerous high-end outliers. Statistical analyses were performed with untransformed data. *, Student's *t* test, *P* < 0.05; **, Student's *t* test, *P* < 3 × 10^−4^.

Initial attempts to determine the time to maximal titer from the initial serologic positive result suggested that PCC, DC, and CM cases developed maximal titers significantly later than PUC cases (Fig. 3A). However, these analyses were revealed to be skewed by numerous outliers in each group (Fig. 3B). When analyzed individually, high-end outliers appeared to represent serologic recurrences, or ≥4-fold (≥2 dilution) increases in titer (see Fig. S1 in the supplemental material). Therefore, a titer-scanning algorithm was developed to detect, to quantify, and to compare serologic recurrences at least 90 days after the initial serologic positive result. Serologic recurrences were detected in all groups but were significantly (Student's *t* test, *P* < 3 × 10^−4^) more common and frequent among PCC, DC, and CM patients, compared with PUC patients (Table 4 and Fig. 3C). Only 9% of PUC patients (22/248 patients) had one or more detectable serologic recurrences, compared with 36% of PCC patients (23/64 patients) (Fisher's exact test, *P* = 5.5 × 10^−7^, compared with PUC patients), 50% of DC patients (43/86 patients) (Fisher's exact test, *P* = 7 × 10^−15^, compared with PUC patients), and 52% of CM patients (19/36 patients) (Fisher's exact test, *P* = 2.7 × 10^−9^, compared with PUC patients). Times to maximal CF titers were then reanalyzed, excluding the 107 patients with serologic evidence of recurrence. The resulting analysis of data for 327 patients revealed no significant difference in times to maximal titers between groups (Fig. 3D). The overall mean time to maximal titer among these patients was 31 days (95% CI, 13 to 50 days). Seventy-six percent of patients (248/327 patients) exhibited maximal titers at the initial serologic diagnosis, and 90% of patients (295/327 patients) developed maximal titers by 50 days postdiagnosis. The remaining 10% of patients (32/327 patients), who developed maximal titers after 50 days, were analyzed further. Sixty-nine percent of those patients (22/32 patients) developed maximal titers at the first serologic test after the initial diagnosis, the schedule of which was uncontrollable, given that all tests were physician ordered. The remaining 10 patients (6 PUC patients, 2 PCC patients, 1 DC patient, and 1 CM patient) all exhibited maximal titers of ≤1:8 and appeared to undergo very minor serologic increases at some point, which did not meet algorithmic recurrence criteria. These data indicate that maximal titers develop usually <30 days, and very rarely >50 days, from initial serologic positivity except in cases of serologic recurrence or therapeutic failure.

**TABLE 4:** Serologic recurrences

Patient group	No. (%) of patients			
	PUC	PCC	DC	CM
With no recurrence	226 (91)	41 (64)	43 (50)	17 (47)
With ≥1 recurrence	22 (9)	23 (36)	43 (50)	19 (52)
*P*^*a*^		5.5 × 10^−7^	7 × 10^−15^	2.7 × 10^−9^

a Fisher's exact test, performed pairwise with control group.

### Serologic improvement rates.

**FIG 4:**
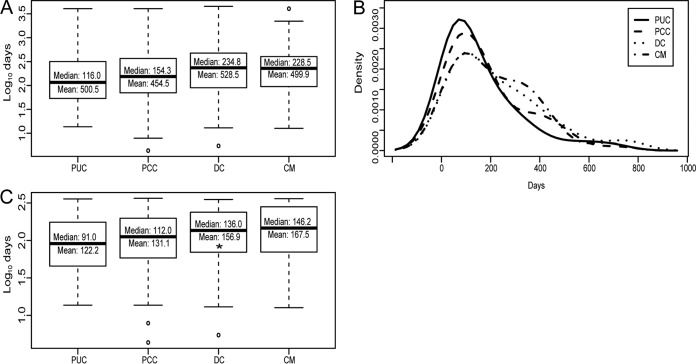
Serologic improvement rates. (A and C) Boxplots showing serologic improvement rates (days per 1-titer [2-fold] reduction) for each group, including serologic nonimprovers (A) and excluding serologic nonimprovers (C). (B) Histogram density plot of serologic improvement rates for each group, indicating that outlier rates of >365 days/dilution were more abundant in complicated (PCC, DC, and CM) cases. Titer data for boxplot analyses were log_10_ transformed to account for numerous high-end outliers. Statistical analyses were performed with untransformed data. *, Student's *t* test, *P* < 0.05.

Serologic improvement rates were then examined to help guide patient and clinician expectations of serologic resolution. Among patients who developed detectable titers, serologic resolution to titers of <1:2 was observed for 56.2% of PUC patients (104/185 patients), 38.3% of PCC patients (23/60 patients), 32.1% of DC patients (27/84 patients), and 48.5% of CM patients (16/33 patients); these differences were not statistically significant (Table 1). Initial analysis of rates of serologic improvement from maximal titers suggested extremely prolonged resolution rates (Fig. 4A), although further analysis revealed that the rates were also skewed by high-end outliers (Fig. 4B). Titer trend analysis of outlier patients indicated that many patients were serologic “nonimprovers” or failed to improve serologically at an overall rate of at least 2-fold (1 dilution) per year (Fig. S2). In total, 15% of PUC patients (36/248 patients), 25% of PCC patients (16/64 patients) (Fisher's exact test, *P* = 0.058, compared with PUC patients), 31% of DC patients (27/86 patients) (Fisher's exact test, *P* = 0.0012, compared with PUC patients), and 25% of CM patients (9/36 patients) (Fisher's exact test, *P* = 0.14, compared with PUC patients) were serologic nonimprovers (Table 5). Reanalysis of serologic improvement rates excluding nonimproving patients revealed that the median overall improvement rate was 91 days/dilution (mean, 122 days/dilution [95% CI, 107 to 138 days/dilution]) for PUC patients, 112 days/dilution (mean, 131 days/dilution [95% CI, 102 to 161 days/dilution]) for PCC patients, 136 days/dilution (mean, 156 days/dilution [95% CI, 129 to 185 days/dilution]) for DC patients, and 146 days/dilution (mean, 168 days/dilution [95% CI, 119 to 216 days/dilution]) for CM patients (Fig. 4C). The only significant difference was between PUC and DC patients, with the latter exhibiting comparatively prolonged titer improvement rates (Student's *t* test, *P* = 0.02). Titer improvement rates of CM patients also appeared prolonged, compared with PUC patients, but this difference was not statistically significant (Student's *t* test, *P* = 0.08). These results indicate that overall titer improvement rates are variable and slightly prolonged in disseminated cases. Importantly, serofast-like phenotypes among PUC patients were surprisingly common and, given the long duration of clinical follow-up monitoring without antifungal therapy in this study, did not appear to affect clinical outcomes.

**TABLE 5:** Serologically nonimproving patients

Patient group	No. (%) of patients			
	PUC	PCC	DC	CM
Improving	212 (85)	48 (75)	59 (69)	27 (75)
Nonimproving	36 (15)	16 (25)	27 (31)	9 (25)
*P*^*a*^		0.058	0.0012	0.14

a Fisher's exact test, performed pairwise with control group.

## DISCUSSION

This, the first quantitative study of coccidioidomycosis serologic dynamics in the antifungal era, reveals new insights that could help guide clinical evaluation and patient management. Physicians often monitor CF titer trends of their patients to help guide treatment decisions but in recent times have done so without established modern serologic criteria for comparison.

Preantifungal studies by Smith et al. (5, 9, 11) indicated that only 2.4 to 9.3% of patients with nondisseminated infections developed maximal titers of >1:16, regardless of clinical manifestations (such as pulmonary residuals or pulmonary cavities), while 58 to 65% of patients with disseminated infections developed titers of >1:16. With this as guidance, CF titers of >1:16 were considered a specific indication of possible dissemination (7). In the present study, not only did patients with uncomplicated pulmonary infections develop significantly higher maximal titers (17% of PUC cases developed titers of >1:16), but also patients with nondisseminated chronic pulmonary infections developed significantly higher titers that largely overlapped with those of CM and DC patients. Thus, higher titers are attainable without dissemination in the antifungal era. Among possible explanations, this could be a related to population changes (e.g., higher susceptible population density in areas in which the fungus is endemic) and/or more severe infections (possibly linked to climate change and increases in airborne arthroconidia [18]).

Accordingly, the classification accuracy of CF was reexamined. Results indicate that the statistically optimal cutoff value for differentiating disseminated cases from nondisseminated cases remains 1:16 but, due to its poor PPV, has dubious clinical utility. In the absence of additional diagnostic testing or clinical guidance, CF titers of ≥1:32 can somewhat reliably raise caution regarding possible complicated infections, including disseminated and chronic infections. However, even this cutoff value has a 16.5% false-positive rate and a 34.4% false-negative rate. Clinical presentation and the presence of risk factors should ultimately have highest priority in decisions involving patient management (1). Table 3 provides sensitivity, specificity, and PPV values for disseminated or complicated infections at each titer threshold and may provide additional guidance.

This study also identified several additional aspects of coccidioidal serologic dynamics that have not been described previously. First, serologic recurrences were observed to be surprisingly common (25% [107/434 patients]), even among patients with uncomplicated infections, and were relatively more common among PCC, DC, and CM patients, compared with PUC patients. Serologic recurrences could hypothetically result from reexposure, granuloma rupture, transient immunologic changes (e.g., from intercurrent illness), or serologic variability, but additional studies are necessary to identify conclusive associations. Further investigation will also be necessary to determine whether such events are clinically relevant or predictable. Second, over 20% of the study population (88/434 patients) had serologic improvement rates exceeding 365 days/dilution, which far exceeded expectations. Anecdotal reports indicated that some patients exhibited serofast-like phenotypes even after years without therapy, but these results indicate that this or related phenomena are much more common than previously appreciated. Importantly, a serofast-like state was significantly more common among patients with increased disease severity and, in many cases, may reflect a host-pathogen equilibrium. For patients with uncomplicated infections, however, all of whom received fluconazole therapy, this phenomenon may indeed reflect a serofast state similar to that occasionally observed in syphilis-infected patients (19). Further studies will be necessary to determine whether these patients are at increased risk of disease recurrence or if they need to be monitored for additional time.

Finally, this study revealed quantitative, actionable, serologic kinetic data. First, times to maximal CF titers were similar across all groups, rarely exceeding 50 days from the initial serologic positive result. Therefore, assuming an incubation period of 10 to 16 days (4) and ≥2 weeks of diagnostic delay, maximal titers likely develop within 2 to 3 months after the initial arthroconidia exposure in most cases. Accordingly, if knowledge of a patient's maximal initial CF titer is desired, then a reasonable recommendation would be to test between 1 and 2 months after initial serologic evidence of coccidioidomycosis is obtained. Second, titer reduction rates were examined and found to range from a median of 3 months/dilution for uncomplicated infections to ≥4 months/dilution for disseminated cases. Notably, since overall titer improvement rates were calculated (i.e., time from maximal titer to subsequent minimum titer), these data may underestimate temporal variations in resolution rates. For example, titer trends in Fig. 1A suggest temporarily accelerated titer improvement rates immediately following initial serologic maxima. This phenomenon is also seen at the individual level in Fig. S1A and D in the supplemental material. However, the resolution rates described offer clinicians some standard against which to compare the serologic responses of their patients and could help inform testing schedules for disease monitoring. For example, a reasonable testing interval to monitor serologic improvement in uncomplicated pulmonary cases is 3 months.

Several limitations are associated with this study. The retrospective nature of the analysis precludes precise measurement of titer development, since the date of symptom onset could not be definitively determined for each patient, and serologic time points were determined by physician order and thus testing was performed at irregular intervals, resulting in significant differences in testing frequency and duration between tests, which could skew kinetic trends. Other limitations of this study include the following. (i) Chart review was performed only between 2009 and 2014, and any subsequent clinical changes that might necessitate case reclassification might not be reflected. (ii) Antifungal drugs and doses and treatment duration and initiation were not controlled for between groups (except that all patients received antifungal therapy and all PUC patients received at least a partial course of fluconazole), which could potentially have implications for disease manifestation and/or CF titer distributions (20). (iii) Comparisons made with preantifungal-era studies could be affected by intervening changes in demographics, environment, climate, and diagnostic reagents. (iv) Only a single reference laboratory was used to acquire CF titer data; therefore, results may not accurately represent the result interrun and intrarun variability or consistency observed in other clinical laboratories. In conclusion, this study provides contemporary assessment of coccidioidomycosis serologic dynamics, revealing many new aspects of disease serology that may aid clinical evaluation and therapeutic decision-making in this antifungal era.

## Supplementary Material

Supplemental file 1
