# Supplementary material for: Coccidioidomycosis Complement Fixation Titer Trends in the Age of Antifungals
Source: J Clin Microbiol. 2018 Nov 27;56(12):e01318-18. doi: 10.1128/JCM.01318-18 (PMC6258849; doi:10.1128/JCM.01318-18)

Figure S1. *Titer Trends of Patients Exhibiting Serologic Recurrences.* Figure S1A-D show titer trends of patients that were algorithmically identified as undergoing at least one serologic recurrence event. Days since initial serologic diagnosis is shown along the x-axis of all panels. S1A shows a PUC patient with 3 recurrences and titer reduction rate of 194 days/dilution. S1B shows a PCC patient with 1 recurrence and a titer reduction rate of 407 days/dilution. S1C shows a PCC patient with 1 recurrence and a titer reduction rate of 48 days/dilution. S1D shows a PUC patient with 1 recurrence and a titer reduction rate of 280 days/dilution. Each circle represents the titer of a single tested serum specimen.

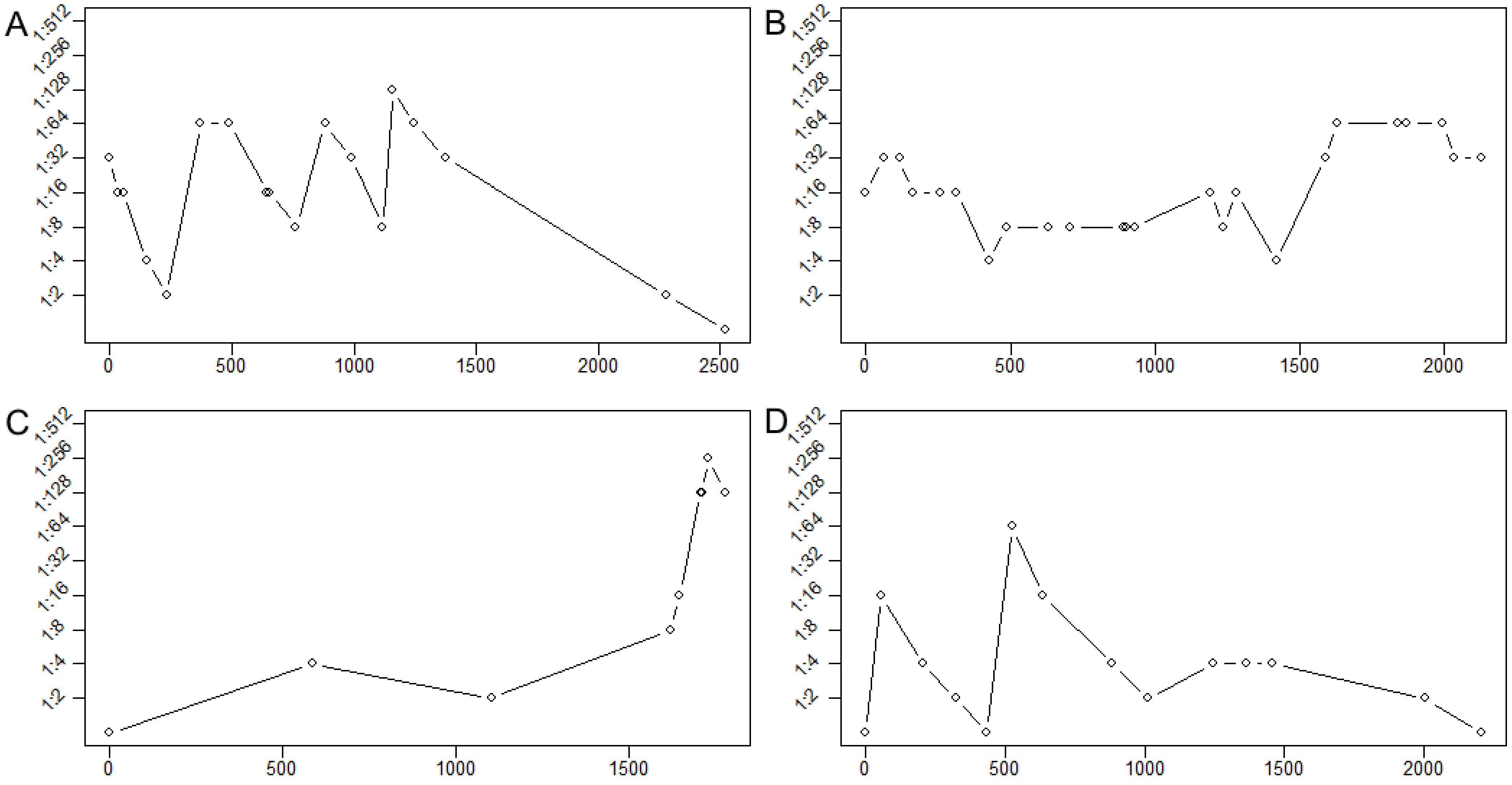

Figure S2. *Titer Trends of Patients Exhibiting Serologic Non-Improvement.* Figure S1A-D show titer trends of patients that were algorithmically identified as failing to improve at a rate of at least 1 titer every 365 days. Days since initial serologic diagnosis is shown along the x-axis of all panels. S2A shows a CM patient with a titer reduction rate of 1962 days/dilution. S2B shows a PUC patient with a titer reduction rate of 954 days/dilution. S2C shows a DC patient with a titer reduction rate of 638 days/dilution. S2D shows a DC patient with a titer reduction rate of 4531 days/dilution. Over 42 years of serologic follow-up are shown for patient S2D. Each circle represents the titer of a single tested serum specimen.

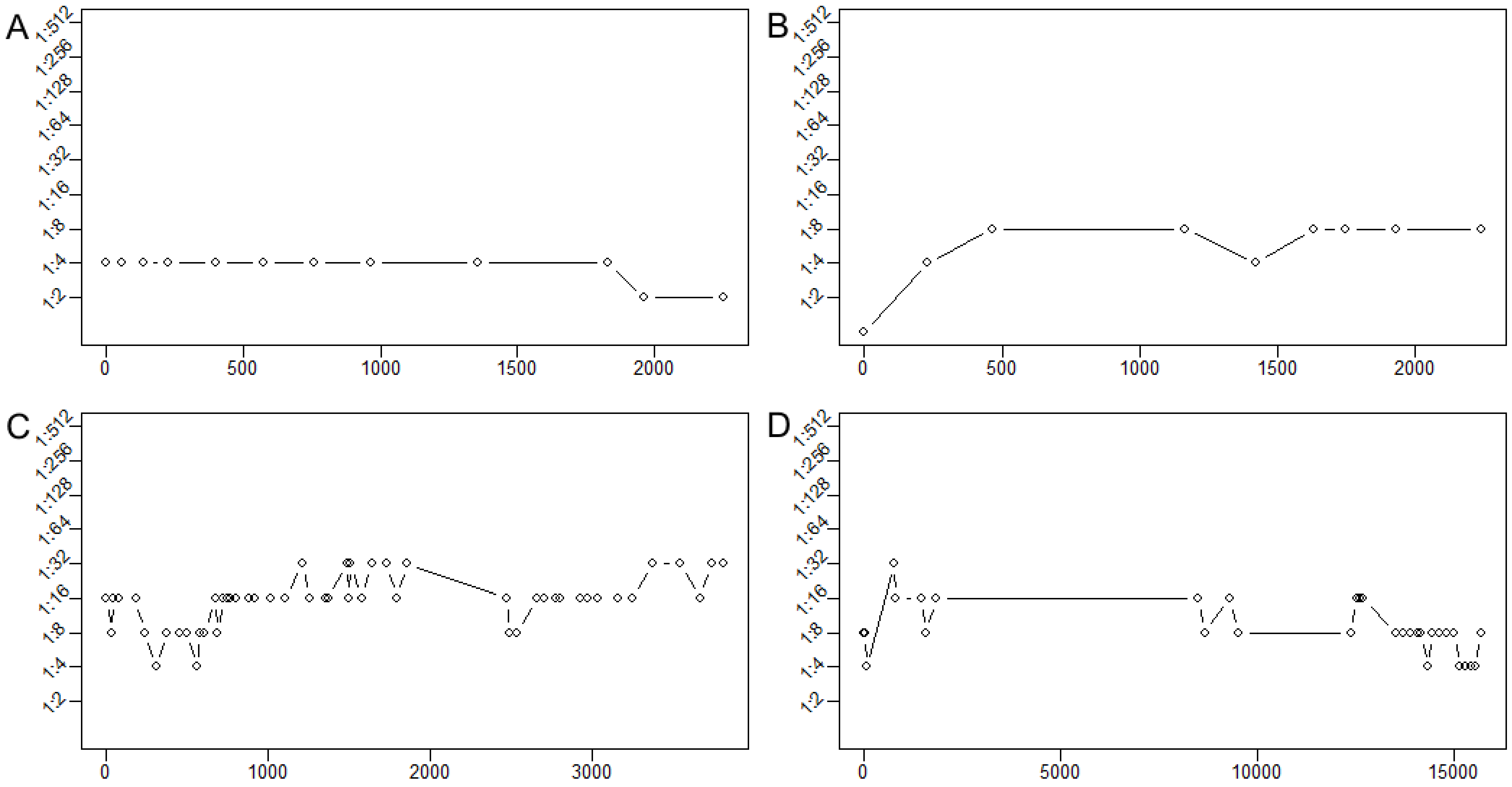

Supplement: Supplemental file 1 [file zjm999096169s1.pdf]
